# HSPD1 Interacts with IRF3 to Facilitate Interferon-Beta Induction

**DOI:** 10.1371/journal.pone.0114874

**Published:** 2014-12-15

**Authors:** Lan Lin, Shan Pan, Jianqing Zhao, Cheng Liu, Pingan Wang, Lei Fu, Xinlin Xu, Meilin Jin, Anding Zhang

**Affiliations:** 1 State Key Laboratory of Agricultural Microbiology, Huazhong Agricultural University, Wuhan, Hubei, China; 2 College of Veterinary Medicine, Huazhong Agricultural University, Wuhan, Hubei, China; 3 College of Agriculture, Liaocheng University, Liaocheng, Shandong, China; Johns Hopkins School of Medicine, United States of America

## Abstract

The production of IFN- I (IFN-α/β) is one of the earliest and most important host-protective responses. Interferon regulatory factor 3 (IRF3) is a critical transcriptional factor in the IFN-β signaling pathway. Although significant progress has been achieved in the regulation of IRF3, the process may be more complicated than previously considered. In the present study, heat shock protein 60 (HSP60, HSPD1) was identified as a novel IRF3-interacting protein. Overexpression of HSPD1 facilitated the phosphorylation and dimerization of IRF3 and enhanced IFN-β induction induced by SeV infection. In contrast, knockdown of endogenous HSPD1 significantly inhibited the signaling pathway. Furthermore, HSPD1 enhanced activation of the IFN-β promoter mediated by RIG-I, MDA-5, MAVS, TBK1 and IKK*ε* but not IRF3/5D, a mock phosphorylated form of IRF3. The present study indicated that HSPD1 interacted with IRF3 and it contributed to the induction of IFN-β.

## Introduction

The innate immune response is an important and evolutionarily conserved mechanism that protects the host against viral infection [1]. The production of IFN- I (IFN-α/β) is one of the earliest and most important host-protective responses [2]. It is induced within hours after infection, modulates immune responses, initiates an antiviral state in cells and is essential for host survival during acute viral infection.

The activation of IFN-I is initiated by the recognition of pathogen-associated molecular patterns (PAMPs) via pattern recognition receptors (PRRs), including the viral RNA sensors RIG-I, MDA-5, LGP2, and DHX33 [3] and the DNA cytoplasmic sensors IFI16, DDX41 and cGAS [4]–[6], among others. Subsequently, the adaptor protein mitochondrial antiviral signaling protein (MAVS, also known as IPS-1/VISA/Cardif) [7], [8] is activated and recruits non-canonical IKK family members, Tank-binding kinase 1 (TBK1) and inhibitor of κB kinase *ε* (IKK*ε*) [9], [10]. Both kinases can phosphorylate IRF-3, resulting in its activation, dimerization and translocation into the nucleus [11]. IRF3 together with other transcription factors assembles on the IFN-α/β promoter to initiate IFN-β transcription in a cooperative manner [12].

Because of the central role in antiviral immune responses, until now, many factors have been identified to interact with proteins in this IFN signaling pathway to promote or suppress the production of IFN-β. For example, TAPE (TBK1-associated protein in endolysosomes) [13] and the mitochondrial targeting chaperone protein 14-3-3*ε* [14] interact with RIG-I to induce IFN-I production. In addition, TRIM14 interacts with MAVS, facilitating the interaction between NEMO and MAVS to enhance virus-induced IFN-I production [15]. In contrast, Mfn2 [16], the proteasome PSMA7 (α4) subunit [17], NLRX1 [18], PCBP2 [19], the tetraspanin protein TSPAN6 [20] and UBXN1 [21] can associate with MAVS to inhibit RLR-induced innate immune responses. Triad3A has been confirmed to interact physically with TRAF3 to negatively regulate signaling [22]. In addition, LUBAC can target NEMO, which is associated with TRAF3, resulting in linear ubiquitination and disrupting the MAVS-TRAF3 complex to inhibit IFN activation [23]. In addition, IFIT3 [24] has been shown to interact with TBK1, leading to enhancement of the signaling pathway. In contrast, TRIM11 [25] interacts with TBK1, resulting in inhibition of the signaling pathway.

IRF3 is a critical transcriptional factor in the IFN-β signaling pathway. Phosphorylation of the Ser385-Ser386, Ser396-Ser398 and Ser402-Thr404-Ser405 clusters by TBK1/IKK*ε* is required to modulate the transformation activation [11]. In addition, phosphorylation of other sites has been shown to be involved in the activation of IRF3 [11], [26], and this process could be directly facilitated by DDX3 and HSP90 [27], [28]. However, IRF3 activation can be negatively regulated by prolylisomerase Pin1, which depends on the polyubiquitination of Pin1 and subsequent proteasome-dependent degradation [29], and this inhibition can be prevented by TRIM21 [30]. In addition, deglutathionylation and ISGylation of IRF3 are also required for its activation [31]–[33]. Although significant progress has been achieved in understanding IRF3 regulation, this process may be more complicated than currently known. Therefore, to better understand this antiviral pathway, further studies of the regulation of IRF3 activation are required.

In the present study, we identified HSPD1 as a novel IRF3-interacting protein. Overexpression of HSPD1 facilitated the phosphorylation and dimerization of IRF3 and subsequently enhanced induction of IFN-β. In contrast, knockdown of endogenous HSPD1 significantly inhibited this signaling. These results indicated that HSPD1could interact with IRF3 and facilitate interferon-beta induction.

## Results

### 1. HSPD1 was identified as an interacting protein of activated IRF3

**Figure 1:**
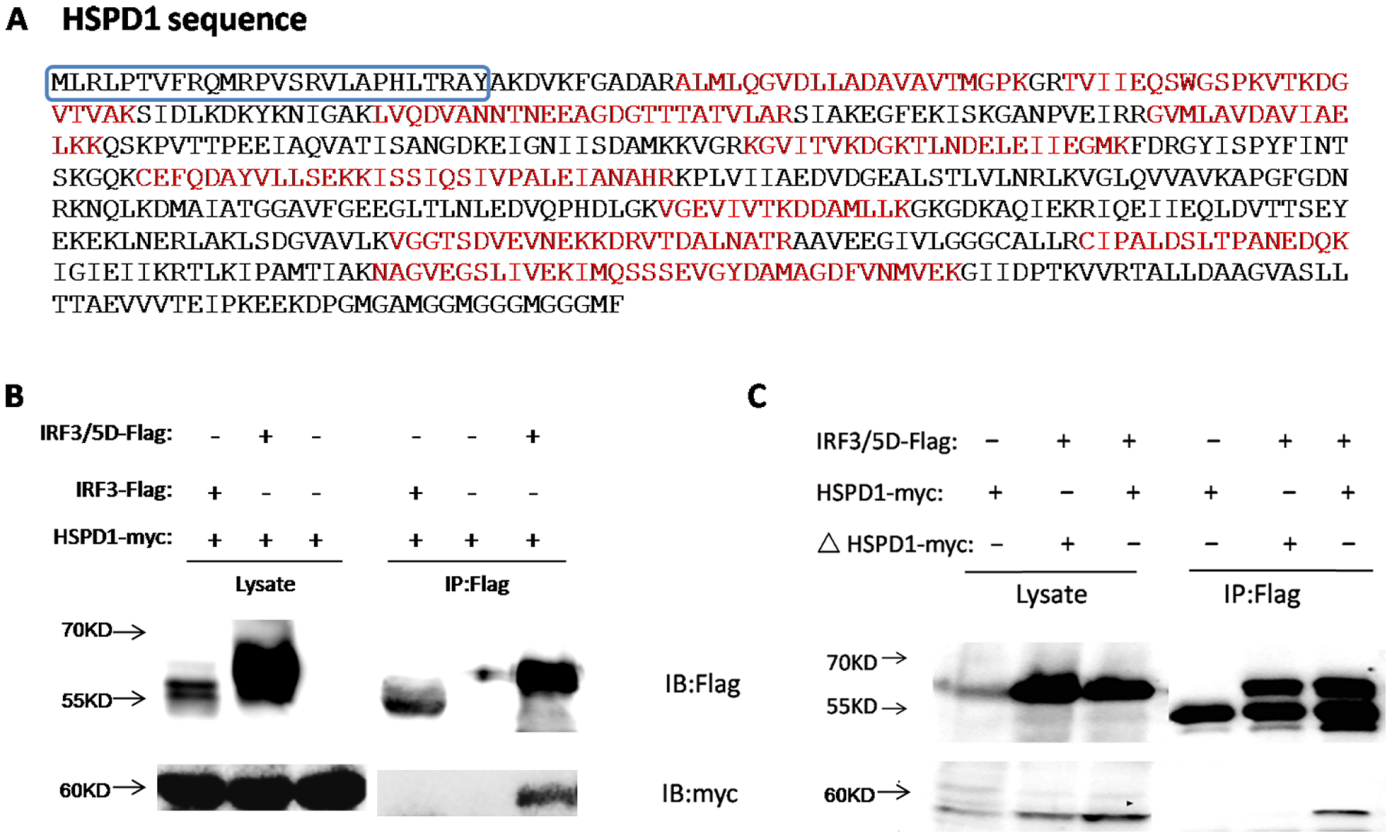
Identification of HSPD1 as an interacting protein of IRF3. **A.** FLAG-tagged IRF3 was exogenously expressed in the HEK293T cell line, and then the cells were activated by overexpression of RIG-IN or mock-transfected with the respective vector. The proteins were extracted and purified with anti-FLAG agarose gel. Next, the purified proteins were analyzed by LC-MS/MS. In comparison with the control sample, 53 peptides and 18 corresponding unique peptides of HSPD1 (coverage: 39.8%) indicated in red were identified in the induced samples. In the blue frame is mitochondrial transit peptide and ΔHSPD1 is lack of the mitochondrial transit peptide. **B.** Myc-tagged HSPD1 was co-expressed with FLAG-tag, FLAG-tagged IRF3, or FLAG-tagged IRF3/5D in HEK293T cells. The proteins were co-precipitated with anti-FLAG agarose gel and then probed with antibodies against Myc-tag or FLAG-tag. **C.** FLAG-tagged IRF3/5D was co-expressed with Myc-tagged HSPD1, Myc-tagged HSPD1 without the mitochondrial transit peptide or control vector in HEK293T cells. The proteins were co-precipitated with anti-FLAG agarose gel and then probed with antibodies against Myc-tag or FLAG-tag.

To better understand the regulation of IRF3 following activation, identification of IRF3-interacting proteins was performed by pull-down assay coupled to LC-MS/MS. FLAG-tagged IRF3 was exogenously expressed in the HEK293T cell line, and then the cells were activated by overexpression of RIG-IN (an active form of RIG-I containing the two CARD domains without the RNA helicase-DEAD box motif) or mock transfected with the respective vector. Whole protein was extracted and then agarose gel-purified using anti-FLAG. The purified proteins from the activation or mock activation were analyzed by LC-MS/MS, and the data was supplied in S1 Figure. Compared with the control sample, among 53 peptides, 18 unique peptides corresponded to HSPD1 (coverage: 39.8%) (**Fig. 1A**). These peptides were identified solely in the activated sample, which indicated that HSPD1 was a potential IRF3-interacting protein during activation.

To further confirm the interaction of IRF3 and HSPD1 following activation, Co-IP assays were performed. HEK293T cells exogenously expressing Myc-tagged HSPD1 with FLAG-tag, FLAG-tagged IRF3, or FLAG-tagged IRF3/5D, which is a constitutively active mock phosphorylated form of IRF3,, were used to extract proteins for purification by agarose gel using anti-FLAG. These Co-IP assays could show whether there was an interaction between IRF3 and HSPD1 following activation. These results demonstrated that Myc-tagged HSPD1 could be co-precipitated with FLAG-tagged IRF3/5D but could not be co-precipitated with FLAG-tagged IRF3 or the control FLAG-tag (**Fig. 1B**). Furthermore, HSPD1 without the mitochondrial transit peptide (**Fig. 1A**) was also overexpressed and another Co-IP assay was performed. And as a result we found that HSPD1 without the mitochondrial transit peptide could not be co-precipitated with FLAG-tagged IRF3/5D (**Fig. 1C**), confirming that the mitochondrial target was necessary for the interaction between HSPD1 and IRF3. In summary, these assays confirmed that HSPD1 protein interacted with IRF3 following activation.

### 2. Co-localization of IRF3 and HSPD1

**Figure 2:**
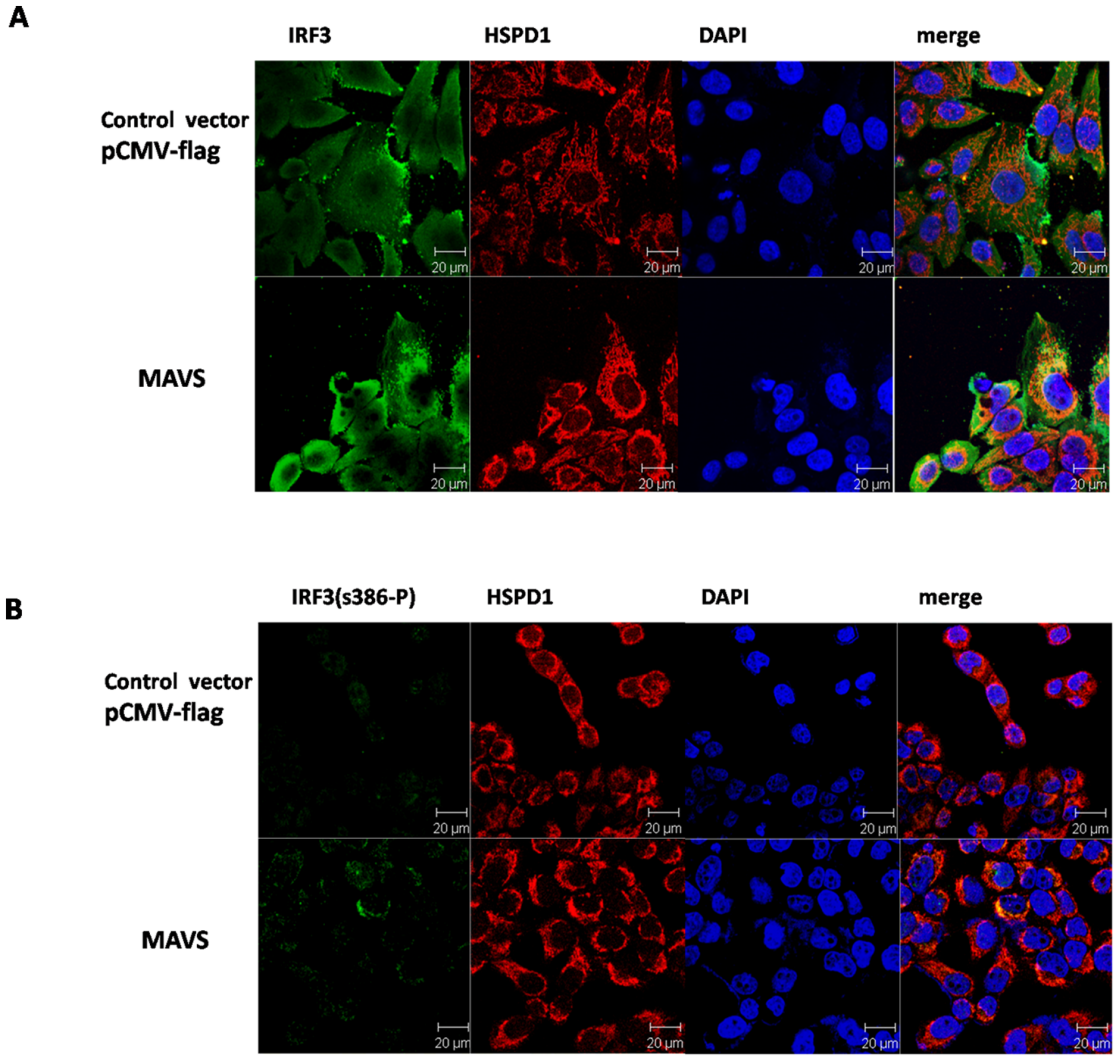
Co-localization of IRF3 and HSPD1. **A.** HeLa cells were transfected with the MAVS or control plasmid. At 8 h post-transfection, the cells were fixed, permeabilized, and then stained with rabbit antibody against IRF3 and mouse antibody against HSPD1 and further incubated with goat anti-mouse IgG H&L (Cy3) and goat anti-rabbit IgG H&L (FITC). Nuclei were stained with DAPI. **B.** HeLa cells were transfected with the MAVS or control plasmid. At 16 h post-transfection, the cells were fixed, permeabilized, and then stained with rabbit antibody against IRF3 (phospho S386) and mouse antibody against HSPD1 and further developed with goat anti-mouse IgG H&L (Cy3) and goat anti-rabbit IgG H&L (FITC). Nuclei were stained with DAPI.

Because HSPD1 interacted with IRF3 in activated but not in resting cells, we investigated to the fate of both proteins after activation using confocal laser microscopy analysis. In resting cells, IRF3 dispersed throughout the cytoplasm while HSPD1 displayed mainly a specific distribution. There was no obvious co-localization of IRF3 and HSPD1 (**Fig. 2A**), which was equivalent to the results in resting cells showing no interaction (**Fig. 1A**). However, when the cells were activated by overexpression of MAVS after 8 h, IRF3 was recruited to HSPD1, and both proteins displayed clear co-localization (**Fig. 2A**). To directly observe the distribution of HSPD1 and activated IRF3, an antibody specific to phosphorylated IRF3 (phospho S386) was used. Not surprisingly, overexpression of MAVS for 16 h resulted in phosphorylation of IRF3. Furthermore, almost all of the activated IRF3 co-localized with HSPD1 at that time point (**Fig. 2B**).

To further show the co-localization of IRF3 with HSPD1, MAVS-BFP was overexpressed as an inducer of IRF3 phosphorylation. It was obvious that phosphorylated IRF3 (phospho S386) co-localized with HSPD1 (yellow arrows) in the cytoplasm of cells which expressed MAVS-BFP (**S2 Figure**). These assays indicated that IRF3 could be recruited to HSPD1 upon activation.

### 3. Overexpression of HSPD1 facilitated IFN-β induction

**Figure 3:**
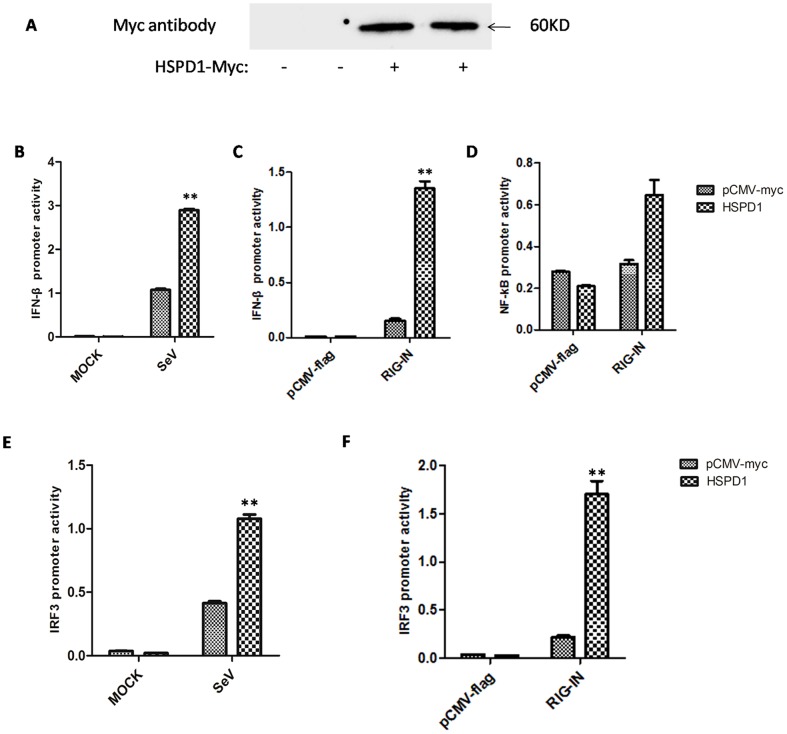
Overexpression of HSPD1 facilitated IFN-β induction. **A.** The expression of Myc-tagged HSPD1 was detected using an antibody against the Myc tag. **B and E.** The HEK293T cells were co-transfected with the luciferase reporter plasmid pIFN-β-Luc or pIRF3-Luc, the *Renilla* luciferase plasmid phRL-TK and plasmid encoding Myc-tagged HSPD1 or control vector. After incubation for 24 h, the cells were infected with SeV or mock-treated with the same buffer. After infection for 8 h, all of the cells were collected and the luciferase activity was measured using a dual-luciferase assay system. Data represent the relative firefly luciferase activity normalized to the *Renilla* luciferase activity. **C and F.** The HEK293T cells were co-transfected with 200 ng of the luciferase reporter plasmid pIFN-β-Luc or pIRF3-Luc, 20 ng of the *Renilla* luciferase plasmid phRL-TK (Promega), 200 ng of plasmid encoding Myc-tagged HSPD1 or control vector, and 200 ng of plasmid encoding RIG-IN or control vector for 36 h. The cells were then collected, and the luciferase activity was measured using a dual-luciferase assay system (Promega) and a luminometer (Turner BioSystems, CA). **D.** The HEK293T cells were co-transfected with 200 ng of the luciferase reporter plasmid p NF-κB -Luc or pIRF3-Luc, 20 ng of the *Renilla* luciferase plasmid phRL-TK (Promega), 400 ng of plasmid encoding Myc-tagged HSPD1 or control vector. After incubation for 24 h, the cells were infected with SeV or mock-treated with the same buffer. After infection for 8 h, all of the cells were collected and the luciferase activity was measured using a dual-luciferase assay system.

IRF3 is an essential transcriptional factor for IFN-β production. Therefore, to address the functional relevance of the HSPD1-IRF3 interaction, we investigated whether HSPD1 was involved in this signaling pathway. It was well known that infection with SeV (Sendai virus) can activate IRF3 (**Fig. S3**) and then induce IFN-β production. In our assay, SeV infection could effectively activate the IFN-β luciferase reporter (**Fig. 3B**). Interestingly, overexpression of HSPD1 in HEK293 cells (**Fig. 3A**) significantly increased activation of the IFN-β luciferase reporter following SeV infection compared with the control under the same conditions (P<0.01) (**Fig. 3B**). By comparison, overexpression of HSPD1 alone could not increase the induction of IFN-β without SeV infection. Similarly, when the cells were stimulated with RIG-IN, HSPD1 also enhanced IFN-β promoter activity (**Fig. 3C**), however, HSPD1 did not enhance the NF-κB promoter as obviously as IRF3 (**Fig. 3D**). Furthermore, overexpression of HSPD1 enhanced expression of the IRF3/7 luciferase reporter following stimulation by either SeV infection or expression of RIG-IN as well (**Fig. 3E, 3F**). Therefore, these results indicated that overexpression of HSPD1 specifically benefited IFN-β induction induced by SeV or overexpression of RIG-IN.

### 4. Knockdown of endogenous HSPD1 impaired the induction of IFN-β

**Figure 4:**
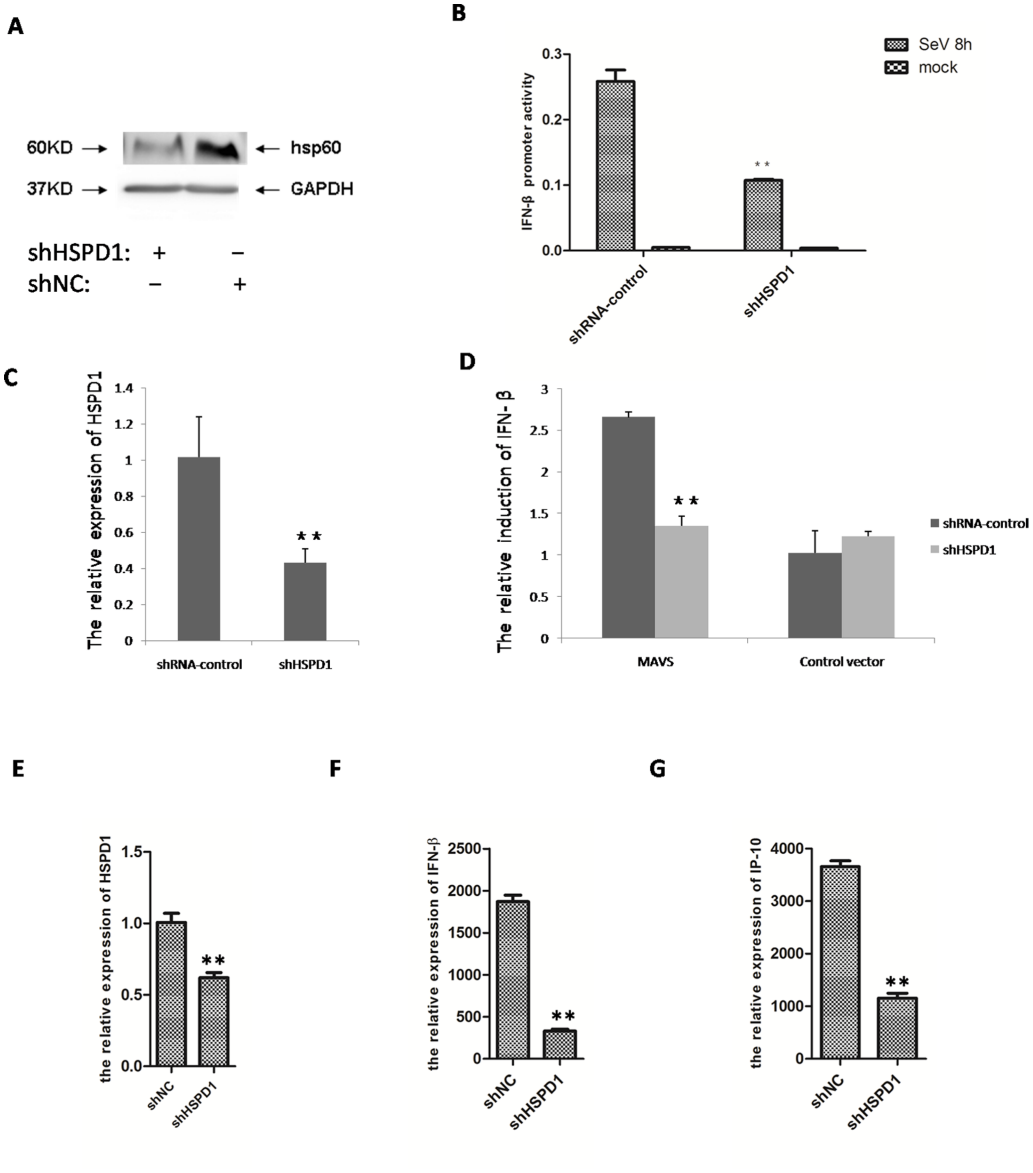
Knockdown of endogenous HSPD1 impaired IFN-β induction. **A.** Hela cells transfected with HSPD1 shRNA or control shRNA showed a significant reduction of HSPD1 expression in cells treated with HSPD1 shRNA compared with control shRNA in a Western blot assay. **B.** SeV infection activated the IFN-β luciferase reporter with control shRNA, and the induction was significantly inhibited with HSPD1 shRNA. **C.** Hela cells transfected with HSPD1 shRNA displayed significant inhibition of the expression of HSPD1 in comparison with control shRNA in a quantitative PCR assay. **D.** Knockdown of endogenous HSPD1 significantly inhibited the induction of IFN-β mRNA induced by overexpression of MAVS for 8 h in a quantitative PCR assay. **E.** HEK293T cells transfected with HSPD1 shRNA displayed significant inhibition of the expression of HSPD1 in comparison with control shRNA in a quantitative PCR assay. **F.** Knockdown of endogenous HSPD1 significantly inhibited the induction of IFN-β mRNA induced by SeV infection for 8 h in a quantitative PCR assay. **G.** Knockdown of endogenous HSPD1 significantly inhibited the expression of IP-10 induced by SeV infection for 8 h in a quantitative PCR assay.

To confirm the functional relevance of the interaction between HSPD1 and IRF3, we used the knockdown approach to assess the function of HSPD1 in IFN-β induction. Effective shRNAs were screened and could reduce the expression of HSPD1 at both mRNA and protein levels (**Fig. 4A, 4C and 4E**). Consistent with previous assays, SeV infection activated the IFN-β luciferase reporter with control shRNA, and this induction was drastically inhibited by knockdown of endogenous HSPD1 (**Fig. 4B**). Furthermore, knockdown of endogenous HSPD1 significantly inhibited the production of IFN-β mRNA induced by overexpression of MAVS for 8 h (**Fig. 4D**) and also inhibited the expression of IFN-β mRNA induced by SeV (**Fig. 4F**). As anticipated, knockdown of endogenous HSPD1 also inhibited the expression of the interferon-stimulated gene IP-10 (**Fig. 4G**). Therefore, these results indicated that knockdown of HSPD1could significantly impair IFN-β induction induced by SeV infection or MAVS induction.

### 5. HSPD1 contributed to the activation of IFN-β signaling

**Figure 5:**
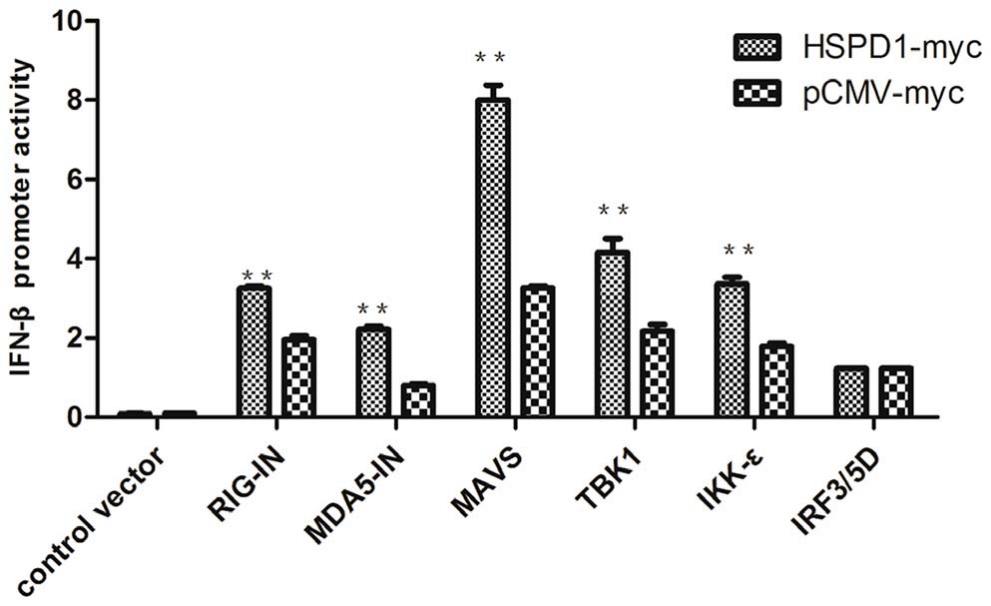
HSPD1 regulated IFN-β induced by various components of the signaling. HEK293T cells were co-transfected with the luciferase reporter plasmid pIFN-β-Luc, the *Renilla* luciferase plasmid phRL-TK, HSPD1 plasmid or control plasmid, and RIG-IN, MDA5-IN, MAVS, TBK1, IKKε, IRF3-5D or control plasmid. At 32 h post-infection, all of the samples were collected and the luciferase activity was measured using a dual-luciferase assay system. Data represent the relative firefly luciferase activity normalized to the *Renilla* luciferase activity.

To further evaluate the function of HSPD1 to activation of IFN-β signaling, we performed a reporter assay to analyze the facilitation of HSPD1 to IFN-β induction by the components of IFN-β signaling. Although overexpression of HSPD1 did not increase IRF3/5D-mediated activation of the IFN-β promoter, it significantly enhanced RIG-IN, MDA-5-IN (an active form of MDA-5 containing the two CARD domains without the RNA helicase-DEAD box motif), MAVS, TBK1 and IKK*ε* mediated activation of the IFN-β promoter (**Fig. 5**). Therefore, HSPD1 could contribute to IFN-β induction by the components of IFN-β signaling.

### 6. HSPD1 facilitated the activation of IRF3 during infection

**Figure 6:**
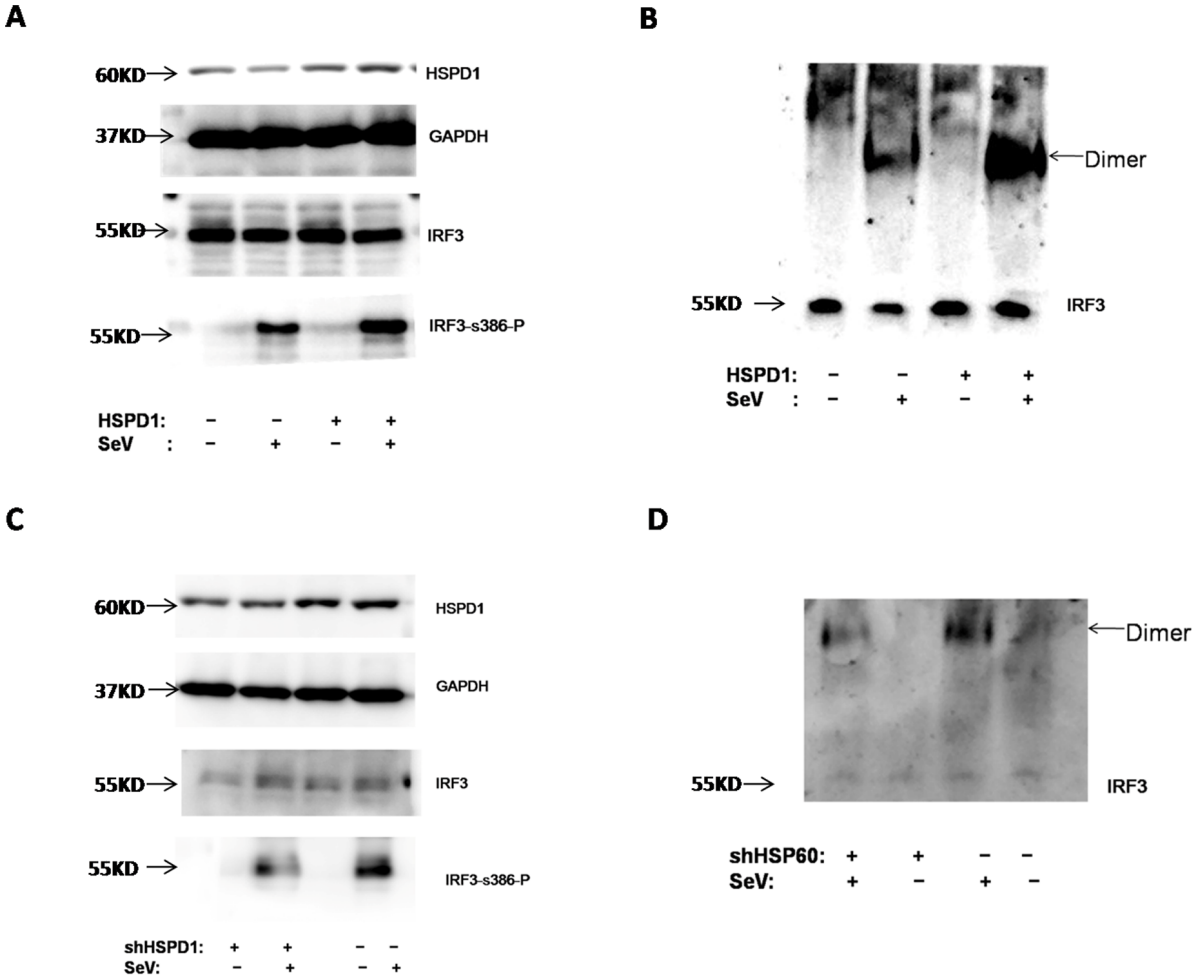
Overexpression of HSPD1 facilitated the activation of IRF3. **A and C.** HEK293T cells were transfected with plasmid encoding Myc-tagged HSPD1 or control vector, shRNA or control vector. After incubation for 24 h, the cells were infected with SeV. At 8 h post-infection, the proteins were extracted and analyzed by SDS-PAGE and further blotted with antibody against IRF3, phospho S386-IRF3 or HSPD1. GAPDH served as an internal control. **B and D.** The proteins were analyzed by native PAGE and then blotted with antibody against IRF3.

Because IRF3 could be recruited and co-localized with HSPD1 following activation, we wanted to know whether HSPD1 facilitated IRF3phosphorylation or not, which is an essential step in IRF3 activation. Consistent with our previous results, SeV infection induced the phosphorylation and then dimerization of IRF3 (**Fig. 6**). Surprisingly, this induction could be significantly enhanced by overexpression of HSPD1 (**Fig. 6A, 6B**). In sharp contrast with this result, knockdown of endogenous HSPD1 clearly inhibited the phosphorylation and dimerization of IRF3 induced by SeV infection (**Fig. 6 C, 6D**). These results indicated that HSPD1 facilitated the activation of IRF3 during its activation.

## Discussion

Heat shock proteins (HSPs) were initially identified as a family of stress-induced proteins characterized by their chaperone activity. Subsequent studies have indicated that the multifunctional proteins play an important role in the regulation of immune responses. As one of the most important proteins, HSPD1 has been reported to be involved in DC maturation, monocyte activation and maturation, B cell activation and Th2 cell polarization through the TLR4 or TLR2 signaling pathway [34], [35]. Cytosolic HSP60 also promotes TNF-α mediated activation of the IKK/NF-κB survival pathway via direct interaction with IKK α/β [36]. In addition, HSPD1 is also reported to be involved in the propagation of several viruses such as hepatitis B virus [37], dengue virus [38], and human cytomegalovirus [39], among others. Therefore, clarification of the roles of HSPD1 in IFN-I antiviral signaling would be helpful to understand the viral pathogenesis. However, until now, few studies have focused on the role of HSPD1 in the IFN-I signaling pathway.

IFN-I signaling plays a central role in the antiviral immune response, and therefore, progress in understanding this pathway not only benefits studies of innate immunity but also contributes to the development of viral pathogenesis. In addition, because IRF3 is a critical transcriptional factor in this signaling pathway, we wanted to identify novel proteins that participate in IRF3 activation. In the present study, HSPD1 was identified as a special protein that could interact with activated IRF3 but did not interact with IRF3 in resting cells. This interaction could be confirmed using a Co-IP assay with IRF3/5D, which is a constitutively active phosphorylated form of IRF3.

In accordance with the results obtained for the interaction between HSPD1 and IRF3, IRF3 did not co-localize with HSPD1 in resting cells, but it was recruited to HSPD1 upon activation at which time the two proteins co-localized. Furthermore, almost all of the activated IRF3 co-localized with HSPD1, at least at the tested time-point following activation by over-expression of MAVS. As shown in Fig. 2, the interaction between HSPD1 and phosphorylatd IRF3 only occurred in the cytoplasm but not in the nucleus, as we could easily detect the green fluorescence of phosphorylated IRF3 but could not find the red fluorescence of HSPD1 in the nucleus. These results clearly indicated that the signaling activation could result in a redistribution of IRF3 to HSPD1, which might play a role in the activation of IRF3 in the cytoplasm but not in its translocation.

To understand the effect of this interaction on IRF3 activation, we investigated the effects of HSPD1 on the induction of IFN-β by performing an IFN-β promoter luciferase reporter assay and real-time fluorescent quantitative PCR. In these assays, overexpression of HSPD1 enhanced IFN-β induction induced by SeV infection or expression of MAVS or RIG-IN. In contrast, knockdown of endogenous HSPD1 could inhibit this signaling. To further confirm that the effect of HSPD1 on IFN- β induction, we further tested the effect of HSPD1 on the IRF3-luc reporter activity induced by SeV or over-expression of RIG-IN, and a similar enhancing activity was observed. Subsequently, we performed an IFN-β promoter luciferase reporter assay to evaluate the effect of HSPD1 on this signaling pathway. We discovered that HSPD1 enhanced activation of the IFN- β promoter in a manner mediated by RIG-I, MDA-5, MAVS, TBK1 and IKK*ε* and not by IRF3/5D. That means HSPD1 could contribute to IFN-β inducted by various components of IFN-β signaling.

Furthermore, we showed that HSPD1 significantly enhanced or inhibited phosphorylation and then dimerization of IRF3 induced by SeV infection in overexpression or knockdown assays, respectively. These results indicated that HSPD1 could facilitate the activation of IRF3, and then benefit IFN-β induction.

Taken together, our results indicated that HSPD1 interacted with IRF3 and contribute to interferon-beta induction following activation. This regulation is new and potentially important. Further studies are needed to elucidate the mechanism by which HSPD1 facilitates the IFN-β signaling.

## Materials and Methods

### 1. Reagents

Antibodies against HSPD1 (mouse original, ab3080), IRF3 (rabbit original, ab76367), and phospho S386-IRF3 (rabbit original, ab76493) were purchased from Abcam, HK. Anti-myc McAb (rabbit original, 9402S), anti-flag McAb (rabbit original, 2368S) were from Cell Signaling Technology. Anti-Flag antibody (M2, mouse) and the ANTI-FLAG M2 Affinity Gel were from Sigma. Anti-GAPDH was from Cali-Bio (mouse original, CB100127). The secondary antibodies goat anti-rabbit Fc-HRP 4041-05 and goat anti-mouse Fc-HRP 1033-05 were from SouthernBiotech. The secondary fluorescence-labeled antibodies goat anti-rabbit IgG H&L (FITC, ab6717) and goat anti-mouse IgG H&L (Cy3, ab97035) were from Abcam. The 4–6-diamidino-2-phenylindole-dihydrochloride (DAPI) was from Invitrogen.

### 2. Plasmids

FLAG-tagged IRF3/5D, IKK*ε* (pcDNA3.0 IKK*ε*), and MAVS were gifts from Rongtuan Lin (Department of Medicine, McGill University). RIG-IN (pEF-Flag RIG-I-N) and p125-Luc (IFN-β-Luc) were kindly provided by Takashi Fujita (Tokyo Metropolitan Institute of Medical Science, Tokyo, Japan). The target sequence of pIRF3- Luc plasmid is TAGGAAAACTGAAAGGGAGAAGTGAAA. TBK1 (pEF-BOS huTBK1 Flag-His) was a gift from Kate Fitzgerald (University of Massachusetts).

FLAG-tagged IRF3 was the WT type control for FLAG-tagged IRF3/5D. The human *hspd1* gene was cloned from 293T cells with the forward primer (5′- AAAGGATCCATGCTTCGGTTACCCACAG) and the reverse primer (5′-AAAAAGCTTTTAGAACATGCCACCTCCC) and then ligated intopCMV-c-myc (Stratagene). HSPD1 without mitochondrial transit peptide was amplified using the forward primer (5′- AAAGGATCCATGCCAAAGATGTAAAATTTG) and the reverse primer (5′-AAAAAGCTTTTAGAACATGCCACCTCCC). MAVS was linked to pBFPN1.

### 3. HPLC-MS/MS shotgun analysis of proteins that interact with IRF3 following its activation

The plasmid encoding FLAG-tagged IRF3 was transfected into the HEK293T cell line for 24 h. Next, the cells were further activated by transfection with vector encoding RIG-IN or by mock transfection with control vector. After activation for 24 h, the cells were lysed in 1 ml of lysis buffer (20 mM Tris [pH 7.5], 150 mM NaCl, 10% glycerol, 1% Triton-X 100, 1 mM DTT, 1 mM PMSF, 40 mM b-glycerol phosphate, 1 mM sodium orthovanadate, protease inhibitor, 5 mM sodium fluoride,10 mM N-ethylmaleimide). Subsequently, the samples were incubated with 30 µl of FLAG-antibody (M2) agarose (sigma) for 2 h at 4°C. After washing 5 times with 0.5 ml of lysis buffer, the precipitated proteins were eluted with 3X FLAG buffer.

The eluate was incubated with 10 mM DL-dithiothreitol (DTT) for 1 h at 37°C and then with 50 mM iodoacetamide in the dark for 40 min. Subsequently, the eluate buffer was changed to 25 mM NH_4_HCO_3_ using Amicon CentriplusYM-3 centrifugal filter devices (Millipore, USA) with a 3-kDa molecular weight cut-off. The protein mixtures were digested with trypsin at 37°C for 20 h and then dried completely using a SpeedVac. Next, the dried peptide samples were redissolved in 0.1% formic acid and injected onto a Zorbax 300SB-C18 peptide trap (Agilent Technologies) where they were desalted with 0.2% formic acid for 20 min. The peptides were eluted from the trap and separated on a reversed-phase C18 column (0.15 mm×150 mm, Column Technology) with a linear gradient of 4% to 100% mobile phase B (0.1% formic acid-84% acetonitrile) in mobile phase A (0.1% formic acid) over a 70-min period. LC-MS/MS measurements were conducted with a linear trap quadrupole (LTQ) mass spectrometer (Thermo Finnigan) equipped with a microspray source. The LTQ mass spectrometer was operated in data-dependent mode with the following parameters: a spray temperature of 200°C and a full scan m/z range from 350–1800. The LC-MS system was fully automated and under the direct control of an Xcalibur software system (Thermo Finnigan). The twenty most intense ions in every full scan were automatically selected for MS/MS.

The MS/MS data were used to search the NCBI database using BIOWORKS software (Version 3.1; Thermo) based on the SEQUEST algorithm. Matched peptide sequences were required to pass the following filters for provisional identification: a delCN value of 0.1 was required for matches, and cross-correlation scores of matches had to be greater than 1.9, 2.2, and 3.75 for the charged state of 1, 2, and 3 peptide ions, respectively.

### 4. Co-immunoprecipitation

The plasmid encoding Myc-tagged HSPD1 was transfected into HEK293T cells with a plasmid encoding FLAG-tagged IRF3, FLAG-tagged IRF3/5D or control vector. After a 24-h transfection, the cells were lysed in 500 µl of lysis buffer. Next, the samples were precipitated with 30 µl of FLAG-antibody (M2) agarose for 2 h at 4°C. After washing with lysis buffer, the proteins were eluted in 50 µl of Laemmli buffer. The pre-precipitated samples and precipitated samples were analyzed by SDS-PAGE followed by blotting with antibody against the Myc-tag or FLAG-tag. Co-immunoprecipitation of Myc-tagged HSPD1 without the mitochondrial transit peptide and FLAG-tagged IRF3/5D was also performed in the present study.

### 5. The effect of overexpression of HSPD1 on IFN-β induction

HEK293T cells were seeded in 24-well plates and then co-transfected with 200 ng of the luciferase reporter plasmid pIFN-β-Luc or pNF-κB-Luc or pIRF3-Luc, 20 ng of the *Renilla* luciferase plasmid phRL-TK (Promega), and 400 ng of plasmid encoding Myc-tagged HSPD1 or control vector. After incubation for 24 h, the cells were infected with SeV or mock-treated with the same buffer for 8 h. Alternately, the cells were co-transfected with 200 ng of the luciferase reporter plasmid pIFN-β-Luc or pIRF3-Luc, 20 ng of the *Renilla* luciferase plasmid phRL-TK (Promega), 200 ng of plasmid encoding Myc-tagged HSPD1 or control vector, and 200 ng of plasmid encoding RIG-IN for 36 h. Next, all of the cells were extracted, and the luciferase activity was measured using a dual-luciferase assay system (Promega) and a luminometer (Turner BioSystems, CA). Data represent the relative firefly luciferase activity normalized to the *Renilla* luciferase activity.

### 6. The effect of knockdown of HSPD1 on IFN-β production

Knockdown of HSPD1 was performed in HeLa cells with shRNA targeting the sequence GGAATCATTGACCCAACAAAG. ShRNA targeting the random sequence TTCTCCGAACGTGTCACGT was also transfected as a control. After transfection for 24 h, the cells were further co-transfected with 200 ng of the luciferase reporter plasmid pIFN-β-Luc, 20 ng of the *Renilla* luciferase plasmid phRL-TK (Promega), and 400 ng of plasmid encoding MAVS or control vector for 24 h. All of the cells were extracted, and the luciferase activity was measured using a dual-luciferase assay system (Promega) and a luminometer (Turner BioSystems, CA). Data represent the relative firefly luciferase activity normalized to the *Renilla* luciferase activity. Western blot analysis was employed to detect endogenous expression of HSPD1 using an antibody against HSPD1.

Concomitantly, HeLa cells were co-transfected with 500 ng of plasmid encoding MAVS or control plasmid and 500 ng of HSPD1 shRNA or control shRNA. After transfection for 24 h, total RNA was extracted with TRIzol (Invitrogen) according to the manufacturer's instructions and then reverse-transcribed into cDNA. The expression levels of IFN-β and HSPD1 were detected by quantitative PCR with primers for IFN-β (5′-GATTCATCTAGCACTGGCTGG/5′-CTTCAGGTAATGCAGAATCC), β-actin (5′-ACCACAGTCCATGCCATCAC/5′-TCCACCACCCTGTTGCTGTA-3) HSPD1 (5′-CCAGCCTTGGACTCATT/5′- CAGCCATAGCATCATAACC), and the interferon-stimulated gene IP-10 (5′-GTGGCATTCAAGGAGTACCTC/5′-TGATGGCCTTCGATTCTGGATT) in the presence of SYBR Green (Applied Biosystems) using a fluorescence temperature cycler (ABI Prism 7000 sequence detection system, Applied Biosystems). The fluorescence signals were quantified using the comparative cycle threshold method. The actin mRNA was used as an endogenous control.

### 7. Confocal Microscopy

HeLa cells were grown to 40% confluency in 12-well plates and transfected with 1 µg of MAVS or MAVS-BFP and control vector at a total weight of 1.5 µg. At 8 or 18 h post-transfection, the cells were fixed with 4% paraformaldehyde and further permeabilized with 0.5% Triton X-100. Subsequently, the cells were incubated in 1× PBS/10% normal goat serum/0.3 M glycine to block non-specific protein-protein interactions for 1 h and then with the mouse antibody against HSPD1 (Abcam) and the rabbit antibody against IRF3 (Abcam) or IRF3 (phospho S386) (Abcam) for 2 hours at room temperature. After washing 3 times, the cells were stained with goat anti-mouse IgG H&L (Cy3) (Abcam) and goat anti-rabbit IgG H&L (FITC) (Abcam) for 45 min and then further stained with or without 4–6-diamidino-2-phenylindole-dihydrochloride (DAPI) (Invitrogen) for 15 min. Finally, all of the above samples were visualized using laser scanning confocal microscopy (ZEISS LSM 510 META).

### 8. The effect of HSPD1 on phosphorylation of IRF3 during infection

After overexpression or knockdown of HSPD1 as described before, the cells were infected with SeV for 8 h and then lysed with 100 µl of lysis buffer. These samples were divided into two parts.

Twenty micrograms of the samples were diluted in 2× Laemmli buffer and then subjected to Western blotting using the antibody against IRF3 (5 µg), phospho S386-IRF3 (5 µg) or HSPD1 (5 µg). GAPDH served as an internal control.

Forty micrograms of the samples were diluted in 5× Native Sample Buffer (5.0 ml of glycerol, 25 mg of bromophenol blue, 150 mg of Tris base, deionized water to 10 ml, pH 6.8). The samples were subjected to ExpressPlus PAGE analysis as described in the technical manual. The proteins were then transferred onto NC membrane and blotted with antibody against IRF3 (Abcam).

### 9. Contribution of HSPD1 to RIG-I, MDA-5, MAVS, TBK1, IKK*ε* and IRF3/5D-mediated activation of IFN-β

HEK293T cells were seeded in 24-well plates and then co-transfected with 200 ng of the luciferase reporter plasmid pIFN-β-Luc, 20 ng of the Renilla luciferase plasmid phRL-TK (Promega), 200 ng of RIG-I, MDA-5, MAVS, TBK1, IKK*ε*, IRF3/5D or corresponding control vector, and 200 ng of plasmid encoding Myc-tagged HSPD1 or control vector. After infection for 32 h, all of the cells were collected and luciferase activity was measured using a dual-luciferase assay system (Promega) and a luminometer (Turner BioSystems, CA). Data represent the relative firefly luciferase activity normalized to the *Renilla* luciferase activity.

### 10. Statistical analysis

The data were expressed the means±standard deviations. All of the data were analyzed using Student's *t*-test. P values were derived to assess statistical significance and are indicated in the figure panels. The significance level for all analyses was P<0.05.

## Supporting Information

S1 Figure **Identification of HSPD1 as an interacting protein of IRF3.** **A.** FLAG-tagged IRF3 was exogenously expressed in HEK293T cells, and then the cells were activated by overexpression of RIG-IN. The proteins were extracted and purified in an anti-FLAG agarose gel and then analyzed by LC-MS/MS. **B.** FLAG-tagged IRF3 was exogenously expressed in HEK293T cells, and then the cells were transfected with the respective control vector. The proteins were extracted and purified in an anti-FLAG agarose gel and then analyzed by LC-MS/MS.
(TIF)

S2 Figure **Co-localization of IRF3 and HSPD1.** HeLa cells were transfected with the MAVS-BFP and control vector at a total weight of 1.5 µg. At 16 h post-transfection, the cells were fixed, permeabilized, and then stained with rabbit antibody against IRF3 (phospho S386) and mouse antibody against HSPD1 and further developed with goat anti-mouse IgG H&L (Cy3) and goat anti-rabbit IgG H&L (FITC).
(TIF)

S3 Figure **Phosphorylation of IRF3 was clearly observed during SeV infection.** HEK293T cells were infected with SeV. Fifteen micrograms of each sample was diluted with 2× Laemmli buffer and then subjected to Western blot analysis using antibody against phospho S386-IRF3 (5 µg). GAPDH served as an internal control.
(TIF)
