# Supplementary figures and images for: HSPD1 Interacts with IRF3 to Facilitate Interferon-Beta Induction
Source: PLoS One. 2014 Dec 15;9(12):e114874. doi: 10.1371/journal.pone.0114874 (PMC4266637; doi:10.1371/journal.pone.0114874)

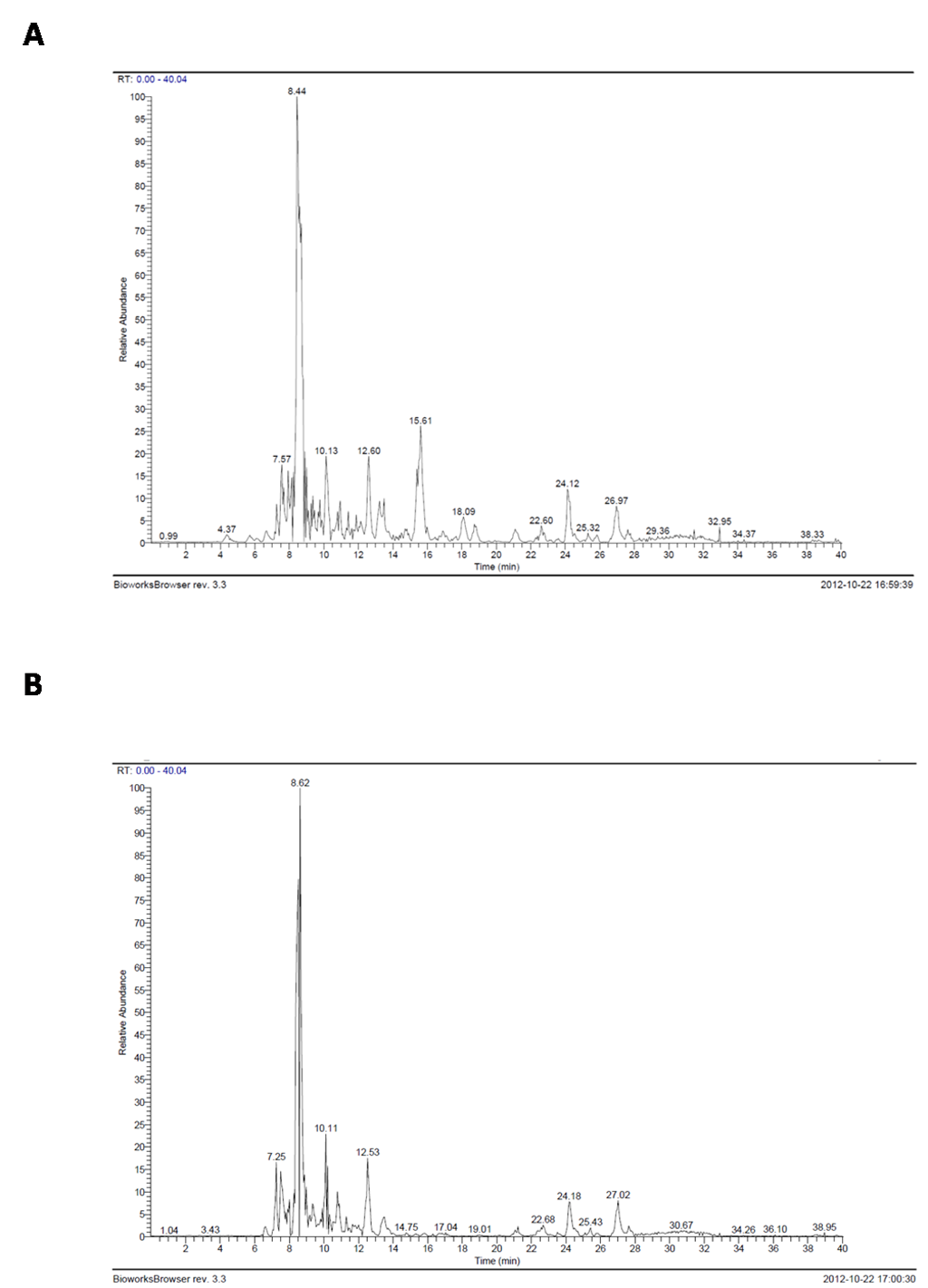

Supplement: S1 Figure — Identification of HSPD1 as an interacting protein of IRF3. A. FLAG-tagged IRF3 was exogenously expressed in HEK293T cells, and then the cells were activated by overexpression of RIG-IN. The proteins were extracted and purified in an anti-FLAG agarose gel and then analyzed by LC-MS/MS. B. FLAG-tagged IRF3 was exogenously expressed in HEK293T cells, and then the cells were transfected with the respective control vector. The proteins were extracted and purified in an anti-FLAG agarose gel and then analyzed by LC-MS/MS. (TIF) [file pone.0114874.s001.tif]

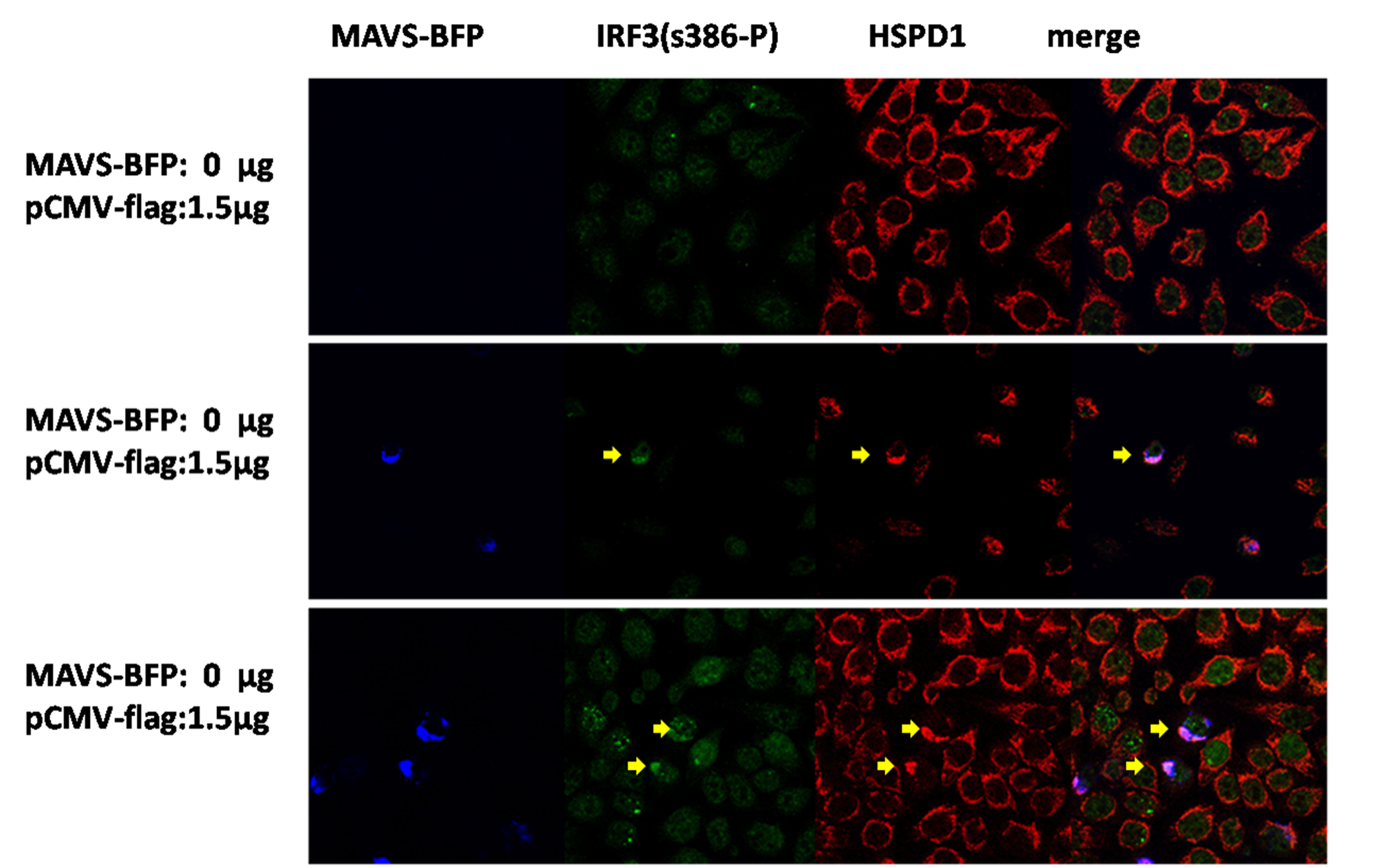

Supplement: S2 Figure — Co-localization of IRF3 and HSPD1. HeLa cells were transfected with the MAVS-BFP and control vector at a total weight of 1.5 µg. At 16 h post-transfection, the cells were fixed, permeabilized, and then stained with rabbit antibody against IRF3 (phospho S386) and mouse antibody against HSPD1 and further developed with goat anti-mouse IgG H&L (Cy3) and goat anti-rabbit IgG H&L (FITC). (TIF) [file pone.0114874.s002.tif]

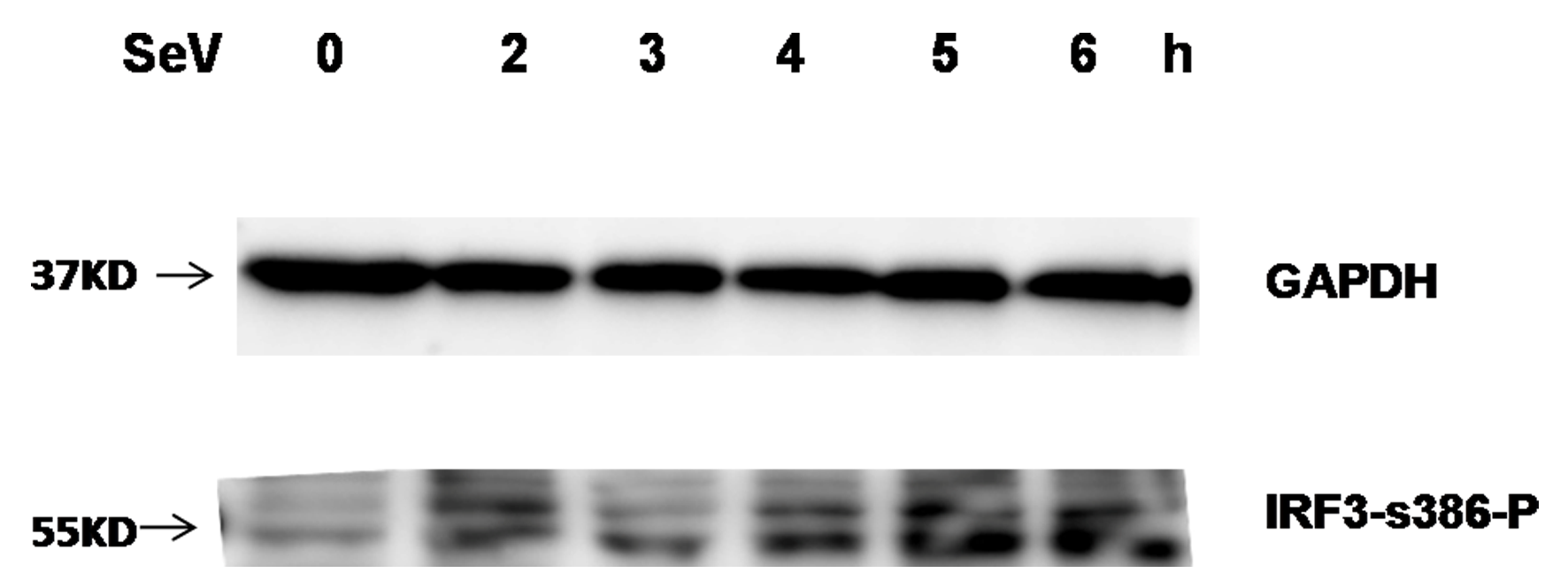

Supplement: S3 Figure — Phosphorylation of IRF3 was clearly observed during SeV infection. HEK293T cells were infected with SeV. Fifteen micrograms of each sample was diluted with 2× Laemmli buffer and then subjected to Western blot analysis using antibody against phospho S386-IRF3 (5 µg). GAPDH served as an internal control. (TIF) [file pone.0114874.s003.tif]
